# Protein Kinase C-Dependent Effects of Neurosteroids on Synaptic GABA_A_ Receptor Inhibition Require the δ-Subunit

**DOI:** 10.3389/fphys.2021.742838

**Published:** 2021-10-25

**Authors:** Erica L. Littlejohn, Carie R. Boychuk

**Affiliations:** Department of Cellular and Integrative Physiology, Long College of Medicine, University of Texas Health San Antonio, San Antonio, TX, United States

**Keywords:** allopregnanolone, inhibition, vagus, PKC, GABA, dorsal vagal complex, dorsal vagal motor neurons, neurosteriod

## Abstract

The dorsal motor nucleus of the vagus (DMV) contains preganglionic motor neurons important for interpreting sensory input from the periphery, integrating that information, and coding the appropriate parasympathetic (vagal) output to target organs. Despite the critical role of hormonal regulation of vagal motor output, few studies examine the role of neurosteroids in the regulation of the DMV. Of the few examinations, no studies have investigated the potential impact of allopregnanolone (Allo), a neuroactive progesterone-derivative, in the regulation of neurotransmission on the DMV. Since DMV neuronal function is tightly regulated by GABA_A_ receptor activity and Allo is an endogenous GABA_A_ receptor ligand, the present study used *in vitro* whole cell patch clamp to investigate whether Allo alters GABAergic neurotransmission to DMV neurons. Although Allo did not influence GABAergic neurotransmission during initial application (5–20 min), a TTX-insensitive prolongment of decay time and increase in frequency of GABAergic currents was established after Allo was removed from the bath for at least 30 min (LtAllo). Inhibition of protein kinase C (PKC) abolished these effects, suggesting that PKC is largely required to mediate Allo-induced inhibition of the DMV. Using mice that lack the δ-subunit of the GABA_A_ receptor, we further confirmed that PKC-dependent activity of LtAllo required this subunit. Allo also potentiated GABA_A_ receptor activity after a repeated application of δ-subunit agonist, suggesting that the presence of Allo encodes stronger δ-subunit-mediated inhibition over time. Using current clamp recording, we demonstrated that LtAllo-induced inhibition is sufficient to decrease action potential firing and excitability within DMV neurons. We conclude that the effects of LtAllo on GABAergic inhibition are dependent on δ-subunit and PKC activation. Taken together, DMV neurons can undergo long lasting Allo-dependent GABA_A_ receptor plasticity.

## Introduction

The efferent motor limb of the parasympathetic nervous system to subdiaphragmatic viscera originates from the brainstem nucleus known as the dorsal motor nucleus of the vagus (DMV). These DMV motor neurons send direct axonal innervations to pancreas, liver, intestine and stomach through the vagus nerve (Berthoud et al., 1991; Browning et al., 1999; Babic et al., 2012). Intact vagal projections to these organs are required for regulation of physiological homeostasis including proper gastrointestinal motility (Ferreira et al., 2001; Travagli et al., 2006) and secretions (Browning and Travagli, 2014), pancreatic secretions (Berthoud et al., 1990; Berthoud and Powley, 1990; Babic et al., 2012; Lamy et al., 2014), hepatic gluconeogenesis (Filippi et al., 2012; Mighiu et al., 2013; Deem et al., 2017), and cardiac function (Machhada et al., 2015, 2017; Moreira et al., 2018). All vagal motor neurons, and DMV neurons specifically, demonstrate considerable GABAergic inhibitory currents (Bouairi et al., 2006; Gao and Smith, 2010a,b), and DMV neurons themselves demonstrate an intrinsic pacemaking property (Browning et al., 1999) making inhibitory GABAergic inputs important to their ultimate output (Babic et al., 2011). Therefore, understanding plasticity of GABAergic signaling to the DMV is critical for both normal DMV neuronal function and overall maintenance of homeostatic regulation (Feng et al., 1990; Washabau et al., 1995; Mussa and Verberne, 2008; Lamy et al., 2014; Shi et al., 2017).

Despite the important role of GABA_A_ receptors in the DMV, we still lack complete understanding of how GABA_A_ receptors are regulated in this nucleus. In particular, little data exist on the role of non-GABA modulators of GABA_A_ receptors and their effects on DMV neurons. One such class of GABA modulators are neurosteroids that result from the catalyzation of either cortisol or progesterone by 5α-reductase. These neurosteroids are traditionally considered secondary endogenous ligands of the GABA_A_ receptor (Ferando and Mody, 2012) and serve as potent allosteric activators of these receptor (Bianchi and Macdonald, 2003). Neurosteroids readily cross the blood brain barrier and recently were shown to concentrate in the brainstem (Sze et al., 2018). Although no studies to date directly assess the action of neurosteroids on DMV neurotransmission and activity, inhibition of 5α-reductase activity does restrain DMV inhibitory GABAergic currents (Littlejohn et al., 2019). Additionally, in the nucleus tractus solaritus (NTS), a nucleus with significant projections to the DMV, neurosteroids influence neurotransmission enough to decrease neuronal activation to excitatory stimuli (Kim et al., 2018). Although the effects of neurosteroids vary significantly across different brain regions (Reddy, 2018), these studies together provide evidence that brainstem circuits and their motor output are regulated by neurosteroids.

Despite limited investigations into the role of neurosteroids in autonomic circuits, they are likely critical to maintaining physiologically relevant signaling. Actions of neurosteroids on GABAergic neurotransmission are directly linked to a variety of brain diseases and disorders, including depression (Maguire, 2019), epilepsy (Reddy, 2011), and anxiety (Longone et al., 2011). Elevated neurosteroid signaling is proposed to dysregulate homeostatic circuits since for example obesity and polycystic ovary syndrome (a syndrome with high risk of weight gain and hyperglycemia) present with high serum concentrations of neurosteroids (Holmberg et al., 2018). Using rodent models, administration of neurosteroids increases food intake and weight gain (Holmberg et al., 2015); while both high fat diet feeding and experimentally-induced diabetes (common animal models for the development of obesity and hyperglycemia) result in increased GABA_A_ receptor neurotransmission to the DMV (Boychuk et al., 2015; Halmos et al., 2015; McMenamin et al., 2018; Clyburn et al., 2019). Therefore, understanding how neurosteroids alter inhibitory GABA_A_ receptor activity in the DMV is critical to understanding the brain’s role in maintaining physiological homeostasis, particularly as it relates to energy homeostatic regulation.

The present study was undertaken then to determine if neurosteroids enhanced inhibitory neurotransmission to decrease neuronal excitability in the DMV. Despite being a progesterone derivative, the neurosteroid, allopregnanolone (Allo), was chosen for the current study for its limited sex differences in *in vitro* signaling (Wilson and Biscardi, 1997). It was hypothesized that Allo induces an increase in inhibitory neurotransmission to DMV neurons, thereby allowing future inhibitory transmission to encode larger inhibition within the brainstem homeostatic regulatory network and decreasing DMV excitability.

## Materials and Methods

All experiments were performed on young adult male C57/Bl6J and GABA_A_ receptor δ-subunit knockout mice (Gabrd^–/–^, δKO; kindly gifted from Dr. Chase Carver (Carver et al., 2014); 25.9 ± 0.37 g; 11.6 ± 0.83 weeks old). All strains were maintained on a C57/Bl6J background. C57/Bl6J mice were purchased from Jackson Research Laboratory and are now an established in-house colony at the University of Texas Health San Antonio (UTHSA). Animals were fed *ad libitum* and housed on a normal 14:10 light cycle. The UTHSA Animal Care and Use Committees approved all animal procedures.

### Electrophysiology

**FIGURE 1:**
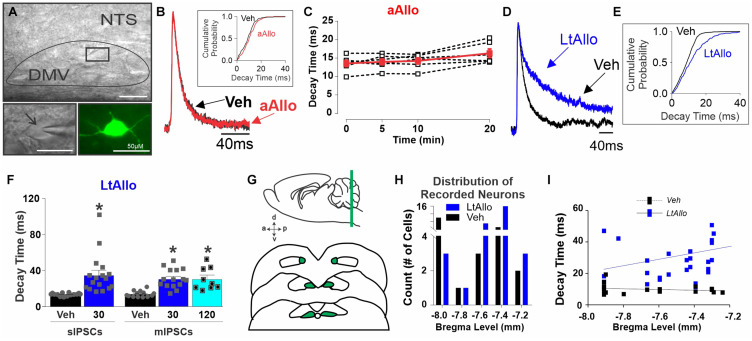
Long-term allopregnanolone (LtAllo) in the dorsal motor nucleus of the vagus (DMV) prolongs phasic γ-aminobutyric acid A (GABA_A_) receptor inhibitory postsynaptic current (IPSC) decay kinetics. **(A)** Top panel: Diffusion contrast imaging of representative DMV neurons with patch pipette. Bottom left panel: Magnification of boxed region in top panel containing a single DMV neuron with patch pipette. Bottom right panel: DMV neuron recovered by biotin-avidin-texas red staining (pseudo-colored green). The scale bar represents 50 μm. Black arrow indicates pipette. **(B)** Averaged trace of spontaneous (s)IPSCs in DMV neurons during Vehicle (Veh, black) and (acute)Allo (aAllo; red, 5 min application) during a paired treatment design. Averaged traces were normalized for amplitude. Inset graph shows a representative sIPSC cumulative probability of decay time during Veh and aAllo (5 min) treatment in a DMV neuron. **(C)** Mean ± SEM of sIPSCs decay time during Veh-application (13.6 ± 0.9 ms) and aAllo-application (100 nM) at 5- (14.0 ± 0.8 ms; *n* = 6 from 3 mice), 10- (14.3 ± 0.8 ms) and 20-min (16.4 ± 1.2 ms; *p* = 0.08) in red. Overlaid points denote individual DMV neuronal responses (black); One-way repeated measure ANOVA. **(D)** An averaged trace of sIPSCs in DMV neurons from a Veh- (black) and a LtAllo-treated (blue) neuron. Averaged traces were normalized for amplitude. **(E)** The representative cumulative sIPSC probability of decay time in DMV neurons from a vehicle-treated (Veh, black) and a LtAllo-treated (blue) neuron. LtAllo shifts the probability to the right. **(F)** Mean ± SEM of IPSCs decay time in Veh- (black bars) and LtAllo-treated (blue bars) neurons. Left panel demonstrates sIPSCs decay time from LtAllo- (35.1 ± 5.3 ms; *n* = 17 from 9 mice) and Veh-treated (13.2 ± 0.4 ms; *n* = 14 from 10 mice) DMV neurons. Right panel is mean ± SEM of IPSC decay time after application of the sodium channel blocker, tetrodotoxin (TTX; mIPSCs). The LtAllo-induced prolongment of decay time in DMV neurons was TTX resistant at 30-min (31.5 ± 2.8 ms; *n* = 15 from 9 mice) and 120-min (31.6 ± 4.1 ms; *n* = 9 from 4 animals) compared to Veh-treated (14.2 ± 1.0 ms; *n* = 13 from 7 mice). Overlaid points denote individual neuronal responses; unpaired Student’s *t*-test for sIPSCs; One-way ANOVA with Tukey’s *post hoc* for mIPSCs. **(G)** Representative diagram illustrates the rostral-caudal distribution of recorded neurons in the DMV. **(H)** Distribution of Veh- (black) and LtAllo-treated (blue) neurons recorded experiments (Veh, *n* = 49; Allo, *n* = 48) plotted as a function of their relative bregma location. **(I)** Correlational analysis of average IPSC decay time across bregma level in Veh-treated (black with dashed line) compared to LtAllo-treated (blue with solid line) neurons. Each square represents a single neuron. Rostrally located DMV neurons correlate more strongly with larger decay times after LtAllo. *Denotes significant difference from Veh. DMV: dorsal vagal motor neurons; NTS: nucleus tractus solitarius. Significance indicates *p* ≤ 0.05.

On the day of experimentation, mice were anesthetized by isoflurane inhalation to effect (i.e., lack of tail pinch response) and decapitated while anesthetized. The brainstem was then rapidly removed and immediately immersed in ice-cold (0–4°C), oxygenated (95% O_2_–5% CO_2_) artificial cerebrospinal fluid (aCSF) containing (in mM) 124 NaCl, 3 KCl, 26 NaHCO3, 1.4 NaH2PO4, 11 glucose, 1.3 CaCl2, 1.3 MgCl2; pH = 7.2–7.4, with an osmolality of 290–305 mosmol/kg H_2_O. aCSF contained the glutamate receptor antagonist kynurenic acid (1 mM; Sigma-Aldrich; St. Louis, MO). The brainstem was mounted on an ice-cold stage, and slices (350 μm) were cut in the coronal plane using a vibratome (Leica Biosystems, Buffalo Grove, IL). Slices containing the dorsal vagal complex (−8.0 through −7.2 mm bregma) were transferred to a holding chamber and incubated in warmed (30–33°C), oxygenated aCSF before being transferred to a recording chamber mounted on the fixed stage of an upright microscope (BX51WI; Olympus, Melville, NY), where they were continually superfused with warmed, oxygenated aCSF. The rostral to caudal bregma distribution of slices used across groups (i.e., Allo or Veh) was similar throughout experiments (Figure 1H).

Whole cell patch-clamp recordings were performed under visual control using infrared illumination and differential interference contrast optics (IR-DIC). For recordings, glass pipettes (2–5 MΩ; King Precision Glass, Claremont, CA) were filled with a solution containing the following (in mM): 130 Cs^+^-gluconate (or K^+^-gluconate), 1 NaCl, 5 EGTA, 10 HEPES, 1 MgCl2, 1 CaCl2, 3 CsOH (or KOH), and 2–3 Mg-ATP, pH 7.2–7.4 adjusted with 5 M CsOH (or KOH). To examine inhibitory postsynaptic currents (IPSCs), voltage clamp mode was used at a holding potential of 0 mV with internal containing Cs^+^ as the primary cation to block K^+^ currents and minimize any influence of K^+^-dependent, postsynaptic GABA_B_ receptors in recorded neurons. Action potential frequency, resting membrane potential (RMP), and input resistance (IR) were measured in current-clamp mode using a K^+^-gluconate internal recording solution. RMP was corrected for liquid junction potential *post hoc* (−7 mV). IR was measured by injecting current-steps (20 pA, 400 ms). To measure action potential response to depolarizing current pulses (400 ms duration) of increasing intensity (from 30 to 210 pA), neurons were clamped at −60 mV, to ensure a consistent starting voltage. The number of action potentials during each depolarizing current step were recorded as a count. All recordings were discarded if series resistance was > 25 MΩ or changed by > 20% throughout the course of the experiment. Mean series resistance was 9.4 ± 0.6 MΩ in Vehicle (Veh)-treated groups and 9.2 ± 0.7 MΩ in all Allo-treated groups. Electrophysiological signals were recorded using an Axoclamp 700B amplifier (Molecular Devices, Union City, CA), acquired at 20 kHz, low-pass filtered at 3 kHz, and stored to a computer using a Digidata 1550B converter and pClamp 10.7 software (Molecular Devices).

### Drugs

Although there are several neurosteroids that are GABA_A_ receptor ligands, Allo was used here since previous reports demonstrated limited sex differences in Allo’s activity at GABA_A_ receptors (Wilson and Biscardi, 1997). Allo ([3α,5α]-3-Hydroxy-pregnan-20-one, Tocris Bioscience; Minneapolis, MN) was initially dissolved in DMSO. This was added to aCSF containing 1 mM KYN for a final working concentration of 100 nM Allo containing 0.1% DMSO. This concentration of DMSO did not affect GABA_A_ receptor-mediated IPSCs, as determined in a series of control experiments (Supplementary Figure 1).

All drugs were applied until steady-state was reached at which time analysis was done. Drugs included the GABA_A_ receptor antagonist bicuculline methiodide (BIC; 30 μM; R&D Systems, Minneapolis, MN), the Na^+^ channel blocker tetrodotoxin (TTX; 1 μM; Alomone Labs, Jerusalem, Israel), the protein kinase C (PKC) inhibitor bisindolylmaleimide I (GFX; 500 nM; Tocris Bioscience), the PKC activator phorbol-12-myristate-13-acetate (PMA; 40 nM; Tocris Bioscience), 4,5,6,7-tetrahydroisoxazolo[5,4-c]pyridin-3-ol hydrochloride (THIP; 3 μM; Tocris Bioscience), an agonist with preference for δ-subunit-containing GABA_A_ receptors. All drugs were dissolved in aCSF containing 1 mM KYN.

### Histology

The location of each recorded cell within the rostral-caudal aspect of the DMV was noted and recovered by staining for biocytin (0.2% added to internal recording solution; Figure 1A). After recording, slices were fixed with 4% paraformaldehyde in 0.01 M phosphate-buffered saline (PBS) overnight at 4°C. Slices were rinsed in PBS and then immersed in avidin-conjugated to Texas red (1:400; Vector Laboratories, Burlingame, CA) in PBS containing 1% Triton X-100 and incubated for 2.5 h at room temperature to identify biocytin-filled neurons (Figure 1A). The anatomical location of each neuron was identified using major neuroanatomical landmarks, including the shape and presence of either the central canal or 4th ventricle and the presence (or absence) of area postrema. These were compared to a known mouse brain atlas for relative location to bregma (Franklin and Paxinos, 2013).

To examine δ-subunit expression in the DMV after Allo application, brainstem slices were taken and incubated as detailed for electrophysiology. After removal from Allo or Veh bath for 30 min, slices were fixed with 4% paraformaldehyde. Serial coronal sections (30 μm) were then taken using a cryostat (Leica Biosystem) and processed for choline acetyltransferase (ChAT) and GABA_A_ receptor δ-subunit. Sections were rinsed with 0.05 M phosphate-buffered saline (PBS; pH 7.4) and non-specific immunoreactivity blocked with 10% normal donkey serum (Jackson Immunoresearch, West Grove, PA) in 0.3% Triton X-100 and PBS. The δ-subunit was identified using a rabbit primary antibody (1:100; Phospho-Solutions; 868A-GDN, Aurora, CO) followed by an Alexa Fluor 488-conjugated donkey-anti-rabbit IgG (1:200; Life Technologies, Carlsbad, CA). ChAT immunoreactivity was identified using a goat primary antibody (1:500; Millipore; AB144P; Burlington, MA) followed by Alexa Fluor 568-conjugated donkey-anti-goat IgG (1:200; Life Technologies). Allo- and Veh-treated tissues were run in parallel with the same antibody cocktails. Image acquisition parameters, including exposure time and intensity, were identical across groups; brightness and contrast in all images were modified together and identically for figure presentation. Negative controls were run without primary antibody. Robust labeling in the cerebellum served as a positive control due to consistently reported high δ-subunit expression in this brain region (Fritschy and Mohler, 1995; Pirker et al., 2000). All imaging was done with an Olympus BX43 microscope, and images were captured with a Retiga R6 (Teledyne Imaging, Tucson, AZ) using filters for the two fluorescent dyes.

### Data Analysis

A minimum of 8–10 min following establishment of whole-cell configuration was used to allow equilibration of the intracellular and recording pipette contents. Following equilibration, spontaneous inhibitory post-synaptic currents (sIPSC) were recorded for 2 min. To determine treatment and drug effects on IPSCs, 2 min of continuous synaptic activity were analyzed offline using MiniAnalysis 6.0.3. (Synaptosoft, Decatur, CA). All events were used to assess IPSC frequency, but only unitary events (i.e., single peak) were used for IPSC amplitude and decay time measurements. For each trace, IPSCs were aligned by their rise and averaged using MiniAnalysis. The average time required for 90–37% of IPSC current to decay is reported as decay time. To determine GABA_A_ receptor-mediated tonic current, BIC was bath applied until a steady state was reached (7–10 min) as previously described (Littlejohn et al., 2019).

For all electrophysiological experiments, only one cell was used per slice, and no more than three cells were used per animal. Data are presented as means ± standard error of the mean (SEM). Statistical measurements were performed using GraphPad Prism 5 (GraphPad Software, La Jolla, CA) and are denoted throughout. Two-tailed unpaired Student’s *t*-tests were used to determine statistical differences when there were only two groups. When variances were unequal, a Welch’s correction was used as noted. To evaluate the distribution of neuronal responses (i.e., the number of neurons responding to ALLO), a non-parametric Mann-Whitney Test was used. A One-way ANOVA with a Tukey’s multiple comparison *post hoc* test was used when there were more than two groups. When variances were unequal, a Kruskal–Wallis test with a Dunn’s multiple comparisons *post hoc* test was used as noted. A One-way or Two-way repeated measures ANOVA with a Sidaks’s multiple comparison *post hoc* test was used when there were more than two groups with repeated measures. Outliers were determined with a robust regression and outlier test (ROUT, *Q* = 0.1%; GraphPad). Probability values ≤ 0.05 were considered significant.

## Results

### Allo Significantly Increased GABA_A_ Receptor Neurotransmission in the Dorsal Motor Nucleus of the Vagus

#### Decay Time of Inhibitory Postsynaptic Currents

The most consistent effect of Allo across multiple brain regions is an increase in the time for GABA_A_ receptor currents to decay (Fancsik et al., 2000; Maguire and Mody, 2009; Ferando and Mody, 2012). Therefore, the following experiments determined if this was true for DMV neurons. In initial experiments during an acute (a)Allo (100 nM) application, mean sIPSC decay time at 5-min (14.0 ± 0.8 ms; *n* = 6 from 3 mice), 10-min (14.3 ± 0.8 ms) or 20-min (16.4 ± 1.2 ms) was not significantly different from sIPSC decay time during vehicle application (time 0; 13.6 ± 0.9 ms; One-way repeated measure ANOVA; *p* = 0.80; Figure 1C). Dose of Allo was based on previous reports (Maguire and Mody, 2007; Abramian et al., 2014).

However, throughout the central nervous system, Allo induces longer lasting changes often associated with its removal (Maguire and Mody, 2007; Abramian et al., 2014; Carver et al., 2014). To determine then if Allo increased GABA_A_ receptor neurotransmission over a longer duration, even after its removal, slices were incubated in either Allo (LtAllo) or Veh (aCSF with 0.1% DMSO) for 30 min before being placed in the recording chamber with aCSF perfusate. DMV neurons were recorded 30–60 min after placement in aCSF perfusate. During LtAllo, sIPSC decay times in DMV neurons from LtAllo-treated slices (35 ± 5.3 ms; *n* = 17 from 9 mice) were significantly increased compared to Veh-treated slices (13 ± 0.4 ms; *n* = 14 from 10 mice; unpaired Student’s *t*-test; *p* = 0.0008; Figure 1F). To determine if this increase in decay time was mediated directly at the GABAergic synaptic contact of DMV neurons, IPSCs were examined in the presence of TTX (1 μM) to block action potential firing (Figure 1F). In TTX, the decay time of miniature (m)IPSCs in DMV neurons from LtAllo-treated slices was again significantly longer (31 ± 2.8 ms; *n* = 15 from 9 mice) compared with DMV neurons from Veh-treated slices (14 ± 0.9 ms; *n* = 13 from 7 mice; One-way ANOVA with Tukey’s *post hoc*; *p* = 0.0001; Figure 1F). Additionally, the effect of LtAllo persisted as far out as was tested (120 min after removal from Allo incubation; 31.6 ± 4.1 ms; *n* = 9 from 4 animals, One-way ANOVA with Tukey’s *post hoc*, *p* = 0.0004; Figure 1F).

DMV neurons were also identified by anatomical location and recovered through biocytin filling and immunohistochemical analysis using avidin-Texas red (Figure 1A; bottom right panel). Anatomical location and electrophysiological properties were compared. There was no correlation between bregma level and phasic current decay time in DMV from Veh-treated slices (*n* = 21, from 15 mice; Spearman correlation; *p* = 0.47, Figure 1I). However, phasic decay time in DMV neurons after LtAllo-treatment (*n* = 30, from 17 mice) significantly correlated with decreasing bregma location, suggesting that DMV neurons located more rostrally were more sensitive to LtAllo (Spearman correlation; *p* = 0.04, Figure 1I).

#### Amplitude of Inhibitory Postsynaptic Currents

The mean amplitude of sIPSC was not different under any Allo exposure duration. After aAllo, the amplitude of sIPSC at 5- (39.2 ± 4.3 pA; *n* = 6 from 3 mice), 10- (38.1 ± 3.7 pA), and 20-min (32.4 ± 3.2 pA) demonstrated no significant difference from time 0 (38.8 ± 3.9 pA; One-way repeated measure ANOVA; *p* = 0.31). DMV neuron sIPSC amplitude from LtAllo-treated slices (27 ± 2.3 pA; *n* = 17 from 9 mice) were also similar to sIPSC amplitudes in Veh-treated slices (30 ± 1.8 pA; *n* = 14 from 10 mice; unpaired Student’s *t*-test; *p* = 0.28). The lack of effect of LtAllo on IPSC amplitude was further confirmed in the presence of TTX since mIPSCs amplitude from LtAllo-treated DMV neurons (21 ± 1.5 pA; *n* = 15 from 9 mice) was also not significantly different from Veh-treated DMV neurons (21 ± 1.2 pA; *n* = 13 from 7 mice, unpaired Student’s *t*-test; *p* = 0.8621).

#### Frequency of Inhibitory Postsynaptic Currents

**FIGURE 2:**
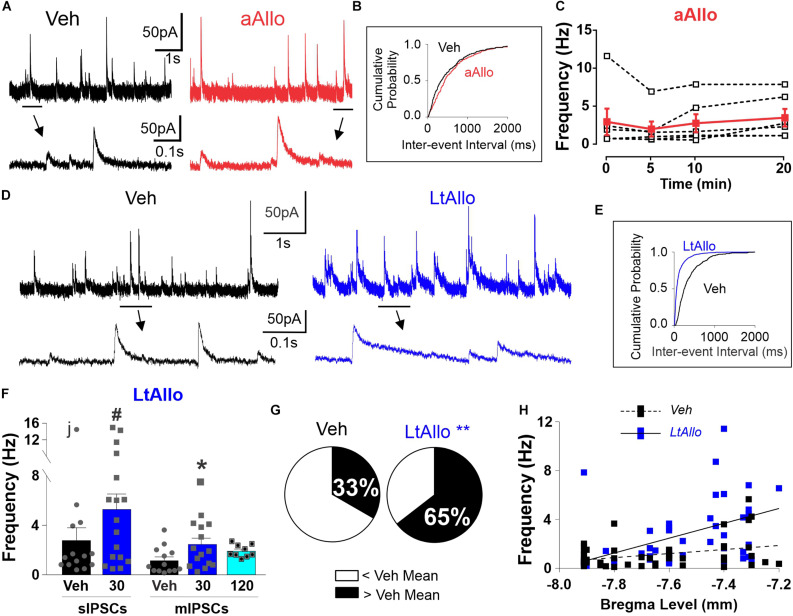
LtAllo in the DMV increases phasic GABA_A_ receptor IPSC frequency. **(A)** Representative trace of sIPSCs from a DMV neuron during Veh (black) and aAllo (red, 5-min application). Bottom panel: magnified section from top trace as indicated by black line. **(B)** Representative sIPSC cumulative probability of inter-event-interval during Veh and aAllo treatment in a representative DMV neuron. **(C)** Mean ± SEM of sIPSCs frequency responses during Veh-application (3.0 ± 1.7 Hz) and aAllo-application at 5- (2.0 ± 1.0 Hz; *n* = 6 from 3 mice), 10- (2.8 ± 1.2 Hz), and 20-min (3.5 ± 1.1 Hz) in red. Overlaid dashed lines denote individual DMV neuronal responses (black); One-way repeated measure ANOVA. **(D)** Representative trace of sIPSCs from a DMV neuron during Veh (black trace) and LtAllo (blue trace). Line and arrow indicate section expanded below. **(E)** Representative sIPSC cumulative probability of inter-event interval (reciprocal of frequency) in a neuron from Veh- and LtAllo treatment. LtAllo left shifts sIPSC cumulative probability. **(F)** Mean ± SEM of IPSCs frequency in Veh- (black bars) and LtAllo-treated (blue bars) neurons. sIPSCs frequency in Veh- (2.8 ± 1.0 Hz; *n* = 14 from 10 mice) and LtAllo-treated (5.4 ± 1.2 Hz; *n* = 17 from 9 mice) neurons. The removal of the neuron labeled with “j” reveals a significantly increased sIPSC frequency after LtAllo. Right-side panel is the mean ± SEM of mIPSC frequency in Veh-treated (1.2 ± 0.3 Hz; *n* = 13 from 7 mice) and Allo-treated (2.5 ± 0.5 Hz; *n* = 15 from 9 mice) neurons, indicating that an increased frequency after LtAllo is TTX resistant. This increase did washout by 120 min after Allo incubation (1.9 ± 0.2 Hz; *n* = 9 from 4 mice). Overlaid points denote individual neuronal responses; j denotes the ROUT identified outlier; unpaired Student’s *t*-test with Welsh’s correction for sIPSC; Kruskal-Wallis with selected Dunn’s *post hoc* for mIPSC. **(G)** Pie graphs illustrating the effect of LtAllo on sIPSC frequency responses expressed as percentage of the total group. Neurons were classified as either greater or less than the mean of Veh-treated frequencies (mean ± SEM of the IPSC frequency of the Veh-treated group). Mann Whitney U test. **(H)** Correlational analysis of averaged IPSC frequency across bregma level in Veh- (black with dashed line) compared to LtAllo-treated (blue with solid line) neurons recorded for this study (Veh, *n* = 49; Allo, *n* = 48). Each square represents a single neuron. ^#^Denotes significant difference from Veh when outlier is excluded, *denotes significant difference from Veh; **denotes significant differences by Mann Whitney *U*-test. Significance indicates *p* ≤ 0.05.

Similar to decay time, initial experiments examining whether aAllo application influenced the frequency of GABAergic currents demonstrated no significant difference in mean sIPSC frequency at any time point examined. During aAllo, mean sIPSC frequency at 5-min (2.0 ± 1.0 Hz; *n* = 6 from 3 mice), 10-min (2.8 ± 1.2 Hz) and 20-min (3.5 ± 1.1 Hz) was similar to sIPSC frequency during time 0 (3.0 ± 1.7 Hz; One-way repeated measure; *p* = 0.60; Figure 2C). Overall, sIPSC frequencies in DMV neurons from Veh-treated slices (2.8 ± 1.0 Hz; *n* = 14 from 10 mice) were also not significantly different compared with neurons from LtAllo-treated slices (5.4 ± 1.2 Hz; *n* = 17 from 9 mice; unpaired Student’s *t*-test with Welsh correction; *p* = 0.10; Figure 2F). However, when data were checked for outliers using a robust regression and outlier test (ROUT, *Q* = 0.1%; GraphPad), a single neuron’s average sIPSC frequency was identified as an outlier in only the Veh group (denoted with an “j” in Figure 2F). With this neuron excluded, sIPSC frequencies from DMV neurons from LtAllo-treated slices were significantly higher compared with neurons from Veh-treated slices (1.9 ± 0.5 Hz; *n* = 13 from 10 mice; unpaired Student’s *t*-test with Welsh correction; *p* = 0.018; Figure 2F). The presence of a small population of DMV neurons that exhibit relatively high frequencies is consistent with previous reports of sIPSC in DMV neurons (Boychuk et al., 2015; Boychuk and Smith, 2016). Therefore, data is presented unaltered. Similar to decay time, the LtAllo-induced increase in IPSC frequency was confirmed to occur at the direct synaptic contact of GABAergic neurons on DMV neurons using TTX. During TTX application, the mean frequency of mIPSCs in DMV neurons from LtAllo-treated slices was significantly higher (2.5 ± 0.5 Hz; *n* = 15 from 9 mice) compared with neurons from Veh-treated slices (1.2 ± 0.3 Hz; *n* = 13 from 7 mice; Kruskal-Wallis with selected Dunn’s *post hoc*; *p* = 0.05; Figure 2F). In slices treated with LtAllo, the number of individual DMV neurons with an average frequency higher than the average Veh value was significantly more (20/32) compared with the number of individual neurons in Veh-treated slices (9/27; Mann-Whitney U test; *p* = 0.04; Figure 2G), providing further confirmation that LtAllo increases IPSC frequency in DMV neurons. Unlike IPSC decay time, the LtAllo-induced increase in frequency washed out by 120 min after removal from Allo incubation (1.9 ± 0.2 Hz; *n* = 9 from 4 mice; Kruskal-Wallis with selected Dunn’s *post hoc*; *p* = 0.12; Figure 2F). Finally, application of the selective GABA_A_ receptor antagonist BIC (30 μM) abolished all sIPSCs between both groups (Supplementary Figure 2C), indicating that LtAllo does not recruit glycinergic IPSCs.

Similar to decay time, anatomical location also influenced the overall effect of LtAllo on IPSC frequency. There was no correlation between bregma level and IPSC frequency in DMV neurons from Veh-treated slices (*n* = 49, from 30 mice; Spearman correlation; *p* = 0.174, Figure 2H). However, IPSC frequency in DMV neurons after LtAllo-treatment (*n* = 48, from 33 mice; Spearman correlation; *p* = 0.001, Figure 1I) significantly correlated with decreasing bregma location, with DMV neurons located more rostrally being more sensitive to the effects of LtAllo on frequency.

#### Tonic GABA_A_ Receptor Currents

Since DMV neurons demonstrate significant GABAergic tonic currents (Gao and Smith, 2010a) and reports from other non-reproductive brain regions indicate that tonic GABAergic currents are increased by neurosteroids (Reddy, 2018), we also examined whether tonic GABA_A_ receptor activity in DMV neurons is altered after Allo exposure. During the aAllo protocol, no consistent change in holding current occurred between Veh (56 ± 14 pA; *n* = 7 from 3 mice) and aALLO application (46 ± 11 pA; paired Student’s *t*-test, *p* = 0.29; Supplementary Figure 2B). To determine differences after LtAllo, we examined GABAergic tonic currents by applying BIC (30 μM) and assessing the differences in holding current from pre-BIC to steady-state BIC application (Supplementary Figure 2C). There was no difference in BIC-sensitive tonic current from LtAllo-treated DMV neurons (43 ± 7.3 pA; *n* = 12 from 8 mice) compared to Veh-treated neurons (32 ± 6.2 pA; *n* = 10 from 7 mice; unpaired Student’s *t*-test; *p* = 0.27). Tonic currents were normalized to cell capacitance (tonic current density) for further analysis. There was no statistical difference in cell capacitance between either treatment group (Vehicle 30 ± 3.6 pF vs. LtAllo 35 ± 3.2 pF; unpaired Student’s *t*-test, *p* = 0.31). BIC-sensitive GABAergic tonic current density from LtAllo-treated DMV neurons (1.3 ± 0.3 pA/pF; *n* = 12 from 8 mice) was not significantly different from current density in DMV neurons from Veh-treated slices (1.1 ± 0.2 pA/pF; *n* = 10 from 7 mice; unpaired Student’s *t*-test, *p* = 0.52; Supplementary Figure 2D). These data suggest that under these experimental conditions Allo does not mediate large changes in GABA_A_ receptor-mediated tonic currents.

### Increased GABA_A_ Receptor Neurotransmission After LtAllo Is Protein Kinase C-Dependent

#### LtAllo Effects on Inhibitory Postsynaptic Current Decay Time Are Protein Kinase C-Dependent

**FIGURE 3:**
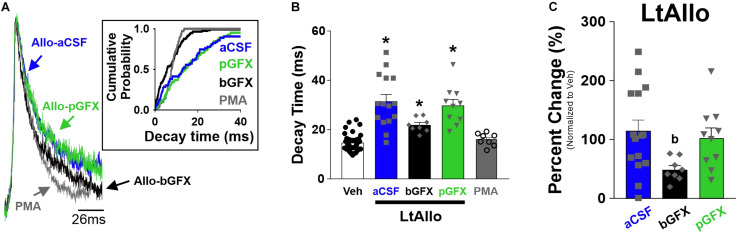
Early protein kinase C (PKC) activation, but not constitutive PKC activity, is required for increased phasic GABA_A_ receptor IPSC decay time during LtAllo. **(A)** Averaged trace of mIPSCs from DMV neurons after LtAllo (Allo-aCSF; blue trace), LtAllo with the PKC inhibitor Bisindolylmaleimide I co-applied in the bath (500 nM; Allo-bGFX; black trace), LtAllo with GFX restricted to the patch pipette (Allo-pGFX; green trace), and bath application of the PKC activator, phorbol 12-myristate 13-acetate in the absence of Allo application (PMA; 40 nM; gray trace). Averaged traces were normalized for amplitude. Inset graph shows mIPSC cumulative probability of decay time in a representative neuron during each treatment. **(B)** Mean ± SEM of mIPSCs decay time during Veh- and LtAllo-treated neurons (duplicated from Figure 1F) during control conditions, Allo-bGFX (21.9 ± 1.0 ms; *n* = 8 from 3 mice), Allo-pGFX (29.79 ± 2.5 ms; *n* = 10 from 7 mice), and PMA (16.0 ± 0.8 ms; *n* = 8 from 6 mice). **(C)** Mean ± SEM of mIPSC decay time relative change (from Veh-treatment group) in Allo-aCSF (114.2 ± 18.8%; *n* = 15 from 9 mice), Allo-bGFX (48.9 ± 6.9%; *n* = 8 from 3 mice), and Allo-pGFX (102.7 ± 17.0%; *n* = 10 from 7 mice). One-way ANOVA with Tukey’s *post hoc*, * indicates significant differences from Veh; (b) indicates significant difference from Allo-aCSF. Significance indicates *p* ≤ 0.05.

In addition to traditional allosteric modulation, previous reports demonstrated that the effects of neurosteroids on GABAergic activity can be mediated through activation of intracellular PKC activity (Fancsik et al., 2000; Abramian et al., 2014). Therefore, we sought to determine if PKC activation was critical to the effects of LtAllo on mIPSC decay time, by either co-incubating slices with the cell permeable PKC inhibitor, GFX, during the 30 min LtAllo incubation period, or including it directly in the patch pipette. When GFX was applied during Veh incubation (regardless of within the bath or pipette), there was no significant difference between decay times compared to control conditions (One-way ANOVA; *p* = 0.81). Therefore, these groups were collapsed into one group. We again repeated the significant increase in mIPSC decay time after LtAllo (31.49 ± 2.76 ms; *n* = 15 from 9 mice) compared to neurons from Veh-treated slices (14.7 ± 0.6 ms; *n* = 40 from 22 mice; One-way ANOVA with Tukey’s *post hoc*; *p* < 0.0001; Figures 3A,B). When compared to the average decay time from Veh-treated DMV neurons, LtAllo increased decay time in DMV neurons by 114.2 ± 18.8% (Figure 3C). When GFX was co-incubated with LtAllo in the perfusate (bGFX), the mean mIPSC decay time was significantly elevated (21.9 ± 1.0 ms; *n* = 8 from 4 mice) compared to decay times from neurons in Veh-treated slices (One-way ANOVA with Tukey’s *post hoc*; *p* = 0.01; Figure 3B). However, this was only a 48.9 ± 6.9% increase compared to the mean mIPSC decay times from neurons in the Veh-treated group, and this was a significant reduction in percent change compared to LtAllo alone (One-way ANOVA with Tukey’s *post hoc*; *p* = 0.03; Figure 3C). When GFX was included in the patch pipette (pGFX), IPSC decay times were again significantly elevated after LtAllo (29.8 ±2.5 ms; *n* = 10 from 7 mice) compared to Veh-treated neurons (One-way ANOVA with Tukey’s *post hoc*; *p* < 0.0001; Figure 3B). However, unlike bGFX, this was a 102.7 ± 17.0% change, which was similar to the percent change seen during LtAllo application (One-way ANOVA with Tukey’s *post hoc*; *p* = 0.85; Figure 3C). Taken together, these data indicate that ∼56% of the effect of LtAllo on decay time is PKC-dependent. They also indicate that although PKC activity contributes to the effect of LtAllo on decay time, a continued elevation in PKC activity is not required. To determine if PKC activation alone was sufficient to mediate an increase in mIPSC decay time, DMV neurons were treated with PMA. mIPSC decay time in DMV neurons from PMA-treated slices (16.0 ± 0.8 ms; *n* = 8 from 5 mice) was not different from mIPSC decay time in DMV neurons from Veh-treated slices (One-way ANOVA; *p* = 0.95; Figure 3B).

#### LtAllo Effects on Frequency Are Protein Kinase C-Dependent

**FIGURE 4:**
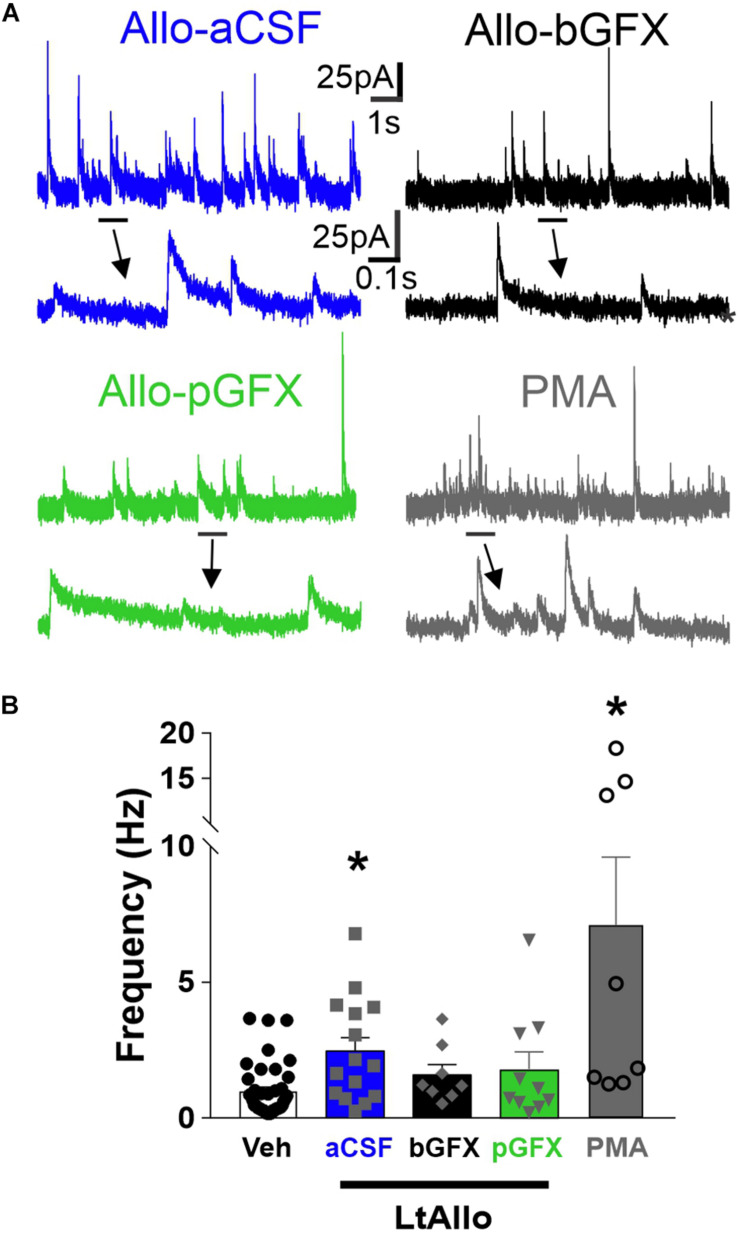
PKC activation is both required and sufficient to increase IPSC frequency during LtAllo. **(A)** Representative mIPSC trace from DMV neurons after Allo-aCSF (blue trace), Allo-bGFX (black trace), Allo-pGFX (green trace), and PMA (gray trace). Line and arrow indicate section expanded below. **(B)** Mean ± SEM of mIPSCs frequency during Veh-treated and Allo-aCSF (duplicated from Figure 2F), Allo-bGFX (1.6 ± 0.4 Hz; *n* = 8 from 3 mice), Allo-pGFX (1.8 ± 0.6 Hz; *n* = 10 from 7 mice), and PMA (7.1 ± 2.5 Hz; *n* = 8 from 6 mice). Kruskal-Wallis ANOVA with Dunn’s *post hoc*, * indicates significant differences from Veh. Significance indicates *p* ≤ 0.05.

Similar to decay time, GFX application (bath or intracellular) did not alter mIPSC frequency under Veh conditions (1.2 ± 0.3 Hz vs. 1.1 ± 0.3 Hz vs. 0.9 ± 0.3 Hz; ANOVA with Tukey’s *post hoc* test; *p* = 0.78), and these groups were again consolidated. The mIPSC frequency in DMV neurons from LtAllo-treated slices (2.5 ± 0.5 Hz; *n* = 15 from 9 mice) were significantly higher compared to mIPSC frequency in Veh-treated slices (1.0 ± 0.2 Hz; *n* = 40 from 22 mice; Kruskal-Wallis ANOVA with Dunn’s *post hoc*; *p* = 0.006; Figures 4A,B). This was a 151.0 ± 50.1% increase from the mean mIPSC frequency of Veh-treated DMV neurons. During bGFX, DMV neuron mIPSC frequency from LtAllo-treated slices (1.6 ± 0.4 Hz; *n* = 8 from 4 mice) was similar to mIPSC frequency in Veh-treated slices (Kruskal-Wallis ANOVA with Dunn’s; *p* = 0.25; Figure 4B). To determine if PKC activation alone was sufficient to mediate the increase in mIPSC frequency after LtAllo treatment, DMV neurons were treated with PMA. When slices were incubated with PMA, DMV neuron mIPSC frequency from PMA-treated slices (7.1 ± 2.5 Hz; *n* = 8 from 5 mice) was significantly increased compared to mIPSC frequency in DMV neurons from Veh-treated slices (Kruskal Willis ANOVA with selected Dunn’s comparisons *post hoc*; *p* = 0.0005; Figure 4B).

### Allo-Induced Protein Kinase C-Dependent Increase in GABA_A_ Receptor Neurotransmission Requires the δ-Subunit of the GABA_A_ Receptor

#### Protein Kinase C-Dependent Effects of LtAllo Require the δ-Subunit

**FIGURE 5:**
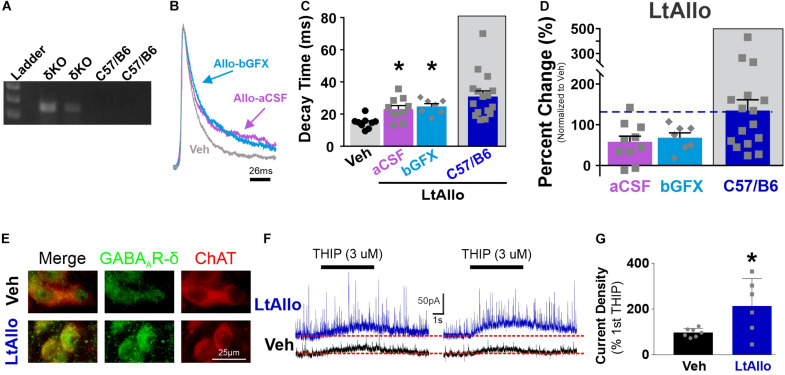
PKC-dependent effects of LtAllo require the GABA_A_ receptor’s δ-subunit. **(A)** Representative genotype from the C57/Bl6J and GABA_A_ receptor δ-subunit knockout mice (Gabrd^–/–^, δKO). **(B)** A representative averaged trace of sIPSCs in a DMV neuron from a δKO mouse during Veh (gray trace), Allo-aCSF (pink trace), and Allo-bGFX (light-blue trace). Averaged traces were normalized for amplitude to emphasize decay time. **(C)** Mean ± SEM of sIPSC decay time in DMV neuron from δKO mice from Veh (14.7 ± 1.2 ms; *n* = 9 from 6 mice), LtAllo (aCSF; 28.53 ± 5.8 ms; *n* = 11 from 4 mice), LtAllo + bGFX (28.7 ± 4.3 ms; *n* = 8 from 3 mice). LtAllo-aCSF in C57/B6 has been duplicated from Figure 1. One-way ANOVA with Tukey’s *post hoc*. **(D)** Mean ± SEM of sIPSC decay time relative change to the average Veh condition decay time in DMV neuron from δKO mice during LtAllo-aCSF (94.1 ± 39.8%; *n* = 11 from 4 mice) and LtAllo-bGFX (95.5 ± 29.3%; *n* = 8 from 3 mice). LtAllo-aCSF in C57/B6 has been duplicated from Figure 1. Unpaired Student’s *t*-test. **(E)** Immunofluorescence microscopy image of the δ-subunit of the GABA_A_ receptor (green) and choline acetyltransferase (ChAT; red) immunoreactivity following from Veh (*n* = 4) or LtAllo (*n* = 4) treated DMV neurons in C57/Bl6 mice. **(F)** Responsivity of the δ-subunit of the GABA_A_ receptor was assessed by 4,5,6,7-tetrahydroisoxazolo[5,4-*c*]pyridin-3-ol hydrochloride (THIP; a δ-subunit agonist) dual-activation in DMV neurons from C57/Bl6 mice. Representative traces demonstrate THIP-inducible current during initial (left panel) and secondary (right panel) picospritzed application of THIP (3μM; 5 s; 20 min interval) in a DMV neuron during Veh (black traces) or LtAllo (blue traces). Change in inducible-current density (pA/pF) was measured as the amplitude difference from baseline steady-state holding current (denoted by dashed line) following the secondary application of THIP relative to the 1st application of THIP, expressed as the percentage. **(G)** Mean ± SEM of change in THIP-inducible current density in DMV neurons from Veh-treated (97.1 ± 7.2%; *n* = 7 from 3 mice) and LtAllo-treated (213.4 ± 48.9%; *n* = 6 from 3 mice). Overlaid points denote individual neuronal responses. Unpaired Student’s *t*-test. *Indicates significant difference from Veh condition. Significance indicates *p* ≤ 0.05.

The influence of neurosteroids is often attributed to interactions with the GABA_A_ receptor’s δ-subunit (MacKenzie and Maguire, 2013). To determine if LtAllo-induced PKC-mediated increases in sIPSC decay time requires the GABA_A_ receptor’s δ-subunit in the DMV, we determined the effect of LtAllo on sIPSCs in slices from δKO mice (Figure 5A). In δKO mice, IPSC decay time was increased following LtAllo (27.0 ± 2.2 ms; *n* = 10 from 4 mice; One way ANOVA with Tukey’s *post hoc*; *p* = 0.0007; Figures 5B,C) compared to neurons from Veh-treated slices (14.7 ± 1.2 ms; *n* = 9 from 6 mice). To determine if the δ-subunit was required for the PKC-dependent effects of LtAllo on decay time in DMV neurons, we tested the effects of GFX bath application on the effect of LtAllo in these mice. In DMV neurons from δKO mice, the average IPSC decay time during bGFX was elevated similar to LtAllo by itself (24.7 ± 1.8 ms; *n* = 8 from 3 mice; *p* = 0.80; Figure 5C). However since only ∼56% of the action of LtAllo is dependent on PKC activation, we again examined the average percent change from Veh conditions. Unlike LtAllo in C57/Bl6 wild type animals that changed ∼114.2%, the average percent change in decay time after LtAllo in the δKO was only 56.7 ± 15.2%. Importantly, the application of GFX during LtAllo did not significantly change this percent increase (68.1 ± 11.9%; unpaired Student’s *t*-test, *p* = 0.59; Figure 5D). Taken together, these data indicate while LtAllo can increase decay time in the absence of PKC and the δ-subunit, the PKC-dependent increases in IPSC decay time of LtAllo require the δ-subunit.

DMV neuron IPSC frequency in Veh-treated (4.7 ± 2.2 Hz; *n* = 9 from 6 mice), LtAllo-treated (2.6 ± 0.4 Hz; *n* = 12 from 4 mice), and LtAllo co-applied with GFX treated slices (3.8 ± 1.3 ms; *n* = 8 from 3 mice) were all similar (Kruskal Willis ANOVA, *p* = 0.69; Figure 5B), suggesting that all the actions of LtAllo on GABAergic frequency in DMV neurons require the δ-subunit.

#### Allo Potentiates Expression of the δ-Subunit

Immunohistochemically labeled δ-subunits associated with the membrane to increase functional expression appear as clustered or enlarged puncta on or close to the surface of the neuron (Mangan et al., 2005; Abramian et al., 2014). Therefore, we qualitatively examined the immunohistochemical staining of the δ-subunit in DMV neurons. In general, somatic staining for the δ-subunit was present, but diffuse in DMV neurons from Veh-treated control mice (*n* = 4; Figure 5E), being distributed lightly throughout the soma. Labeling in the DMV from LtAllo-treated mice (*n* = 4) was characterized by bright immunoreactive puncta in DMV neurons (Figure 5E), which was qualitatively different than what was observed in Veh-treated neurons. These findings imply that increased membrane expression of δ-subunits may be associated with the increased functional expression of IPSCs demonstrated in electrophysiological recordings from DMV neurons in LtAllo-treated neurons.

Previous reports also suggested that a repeated application of a GABA_A_ receptor agonist decreases receptor activity as evidenced by decreasing evoked-amplitude, and that neurosteroid exposure results in increased receptor activity (i.e., increased evoked amplitude), and not decreased (Abramian et al., 2014). Therefore, we tested if LtAllo also potentiates activity of GABA_A_ receptors in DMV neurons. We used an exposure to two brief (5 s) puffs of the δ-subunit agonist THIP (1 μM) separated by 20 min during either Veh application or LtAllo (Figure 5F). After Veh application, the current density of a second THIP application was 97.1 ± 7.2% (*n* = 7 of 3 mice; Figure 5G) of the initial THIP response, representing a general lack of potentiation after activation. However, after Allo administration, the current density of the second THIP application was 213.4 ± 48.7% of the initial THIP response (*n* = 6 of 3 mice) and this was a significant increase compared to Veh-treatment (unpaired Student’s *t*-test; *p* = 0.03). Therefore, consistent with immunohistochemical data, LtAllo significantly increased the functional expression of δ-subunit-containing GABA_A_ receptors at the neuronal membrane, suggesting that LtAllo works to increase the expression of THIP-responsive GABA_A_ receptors.

### LtAllo Decreases Dorsal Motor Nucleus of the Vagus Neuronal Excitability

**FIGURE 6:**
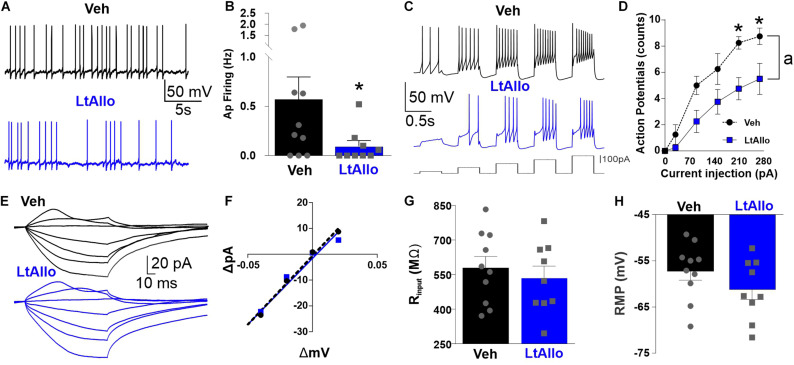
Neuronal excitability of DMV is decreased during LtAllo. **(A)** Representative traces of action potential (AP) firing from Veh- (black trace) and LtAllo-treated (blue trace). **(B)** Mean ± SEM of spontaneous AP firing in Veh- (0.6 ± 0.2 Hz; *n* = 9 from 5 mice) and LtAllo-treated (0.1 ± 0.06 Hz; *n* = 9 from 5 mice) neurons. Unpaired Student’s *t*-test. **(C)** Representative traces showing the response of DMV neurons to step-injections of direct depolarizing current (60 pA-step, 400 ms long) from 30 to 270 pA during Veh- (black trace) and LtAllo-treatment (blue trace). **(D)** Mean ± SEM AP-response curves from Veh-treated (range: 0–8.8 AP; *n* = 4 from 2 mice) and LtAllo-treated (range: 0–5.5 AP; *n* = 4 from 2 mice) DMV neurons during LtAllo. Holding potential, −60 mV. Unpaired Student’s *t*-test. **(E)** Representative traces of membrane response from DMV neurons from Veh- and LtAllo-treated groups to stepped current injections. **(F)** The current-voltage (I–V) relationship graph from representative Veh- and LtAllo-treated DMV neurons. **(G)** Mean ± SEM of IR in Veh- (579.1 ± 55.0 MΩ; *n* = 9 from 5 mice) and LtAllo-treated (535.6 ± 51.3 MΩ; *n* = 9 from 5 mice). Unpaired Student’s *t*-test. **(H)** Mean ± SEM of resting membrane potential (RMP) in Veh-treated (−55.9 ± 1.6 mV; *n* = 9 from 5 mice) and LtAllo-treated (61.2 ± 2.2 mV; *n* = 9 from 5 mice) neurons. Two-way repeated-measures ANOVA, interaction, *p* = 0.02; Sidak’s *post hoc*. (*) Significant difference from Veh condition. (a) Significant difference from Veh and LtAllo regardless of current injection. Significance indicates *p* ≤ 0.05.

To determine if LtAllo significantly decreases the excitability of DMV neurons, DMV neurons were recorded using current clamp configuration. Spontaneous action potential firing frequencies in DMV neurons from LtAllo-treated slices (0.09 ± 0.06 Hz; *n* = 9 from 5 mice) were significantly lower than DMV neurons from Veh-treated slices (0.6 ± 0.3 Hz; *n* = 9 from 5 mice; unpaired Student’s *t*-test; *p* = 0.048; Figures 6A,B). Although there was a strong trend (*p* = 0.07), RMP was not significantly different between LtAllo- (−61.2 ± 2.2 mV) and Veh-treated DMV neurons (−55.9 ± 1.6 mV, unpaired Student’s *t*-test; Figure 6H). There was also no significant difference in IR between either treatment (LtAllo: 535.6 ± 51.2 MΩ vs. Veh: 579.1 ± 55.0 MΩ; unpaired Student’s *t*-test; *p* = 0.57; Figures 6E–G). We further investigated the reduction in excitability using stepped depolarizing current injections. An examination of the relationship between the firing response and the amount of current injected indicated that during LtAllo (*n* = 4 from 2 mice) DMV neurons fire less action potentials compared to Veh-treated neurons (*n* = 4 from 2 mice) at the same intensities (Two-way repeated measures ANOVA with Sidak’s multiple comparisons *post hoc*; Interaction, *p* = 0.023; Figures 6C,D). The difference in action potential response was most prominent at higher current intensities between LtAllo-treated (210 pA: 4.8 ± 0.9 and 270: 5.5 ± 1.2 action potentials) and Veh-treated neurons (210 pA: 8.3 ± 0.5 action potentials; *p* = 0.02 and 270 pA: 8.8 ± 0.6 action potentials; *p* = 0.03).

## Discussion

The present study found that DMV neurons exhibit long-term PKC-dependent increases in GABAergic neurotransmission after exposure to Allo. Specifically, this included a prolonged decay time and increased frequency after LtAllo (but not aAllo) that was mediated by actions directly at the synapse of GABAergic neurons since both parameters were TTX-insensitive. Allo-mediated plasticity was limited to synaptic GABA_A_ receptor activity since tonic currents were not different. The PKC-dependent actions of LtAllo required the δ-subunit of the GABA_A_ receptor, and likely resulted from increased GABA_A_ receptor membrane expression. LtAllo also decreased both spontaneous and evoked action potential firing, suggesting that this increase in GABAergic neurotransmission is sufficient to decrease DMV neuronal excitability. Taken together, these data are the first to demonstrate that DMV neurons have significant PKC-dependent LtAllo-induced increases in GABAergic neurotransmission. Additionally, they demonstrated that PKC-dependent increases in inhibition associated with LtAllo requires the δ-subunit of the GABA_A_ receptor.

To our knowledge, this is the first examination of the role of Allo on inhibitory neurotransmission in the DMV. In DMV neurons, Allo failed to induce a consistent response in any parameter of GABAergic neurotransmission examined after 5–20 min of exposure. Traditionally, neurosteroids serve as allosteric modulators of the GABA_A_ receptor resulting in modifications of GABA_A_ kinetics (i.e., lengthening of decay time) (Haage and Johansson, 1999; Wang, 2011) via pore dilation and increased opening of the receptor’s desensitization gate allowing for increased open time duration (Miller et al., 2017). Allo can also induce immediate phosphorylation of the receptor (Gyenes et al., 1994), but these effects occur rapidly (within 5 min of exposure) (Jovanovic et al., 2004). Therefore, the failure of Allo to induce immediate changes in GABAergic neurotransmission in the DMV indicates that Allo is not likely increasing GABA_A_ receptor activity through immediate changes in receptor pore size and desensitization kinetics by simple allosteric modulation in DMV neurons.

Rather, Allo is likely increasing GABAergic currents through less traditional modulation. In particular, more recent reports identify a unique protein kinase-dependent effect of neurosteroids to enhance GABA_A_ receptor signaling (Connelly et al., 2013). These protein kinase-dependent effects produce long lasting increases in neurotransmission which can occur even after removal of a neurosteroid. The present data provide evidence that GABA_A_ receptor subunits in the DMV confer strong long lasting, PKC-dependent actions of Allo on receptor kinetics since Allo prolonged decay time of GABAergic neurotransmission in DMV neurons 30–120 min after exposure and this effect was abolished after PKC inhibition. Our data further demonstrated that neither PKC activation nor inhibition was sufficient to alter GABA_A_ receptor decay time under Veh conditions. Therefore, in the absence of Allo exposure, the influence of PKC on GABAergic receptor activity is minimal. LtAllo-induced activation of PKC also did not require elevated constitutive PKC activity since restriction of the PKC inhibitor to the patch pipette during recording did not prevent the LtAllo-induced increase in decay time. Taken together, the action of LtAllo on GABA_A_ receptors must in part recruit a PKC intracellular signaling pathway, making the DMV a brain region with strong PKC-dependent modulation of GABA_A_ receptors.

The PKC-dependent effects of LtAllo in the DMV also required the GABA_A_ receptor’s δ-subunit since mice lacking the δ-subunit did not demonstrate PKC-dependent increases in decay time. Although δ-subunits are traditionally considered extra- or peri-synaptic receptors (Nusser et al., 1998; Semyanov et al., 2003), the presence of the δ-subunit does confer increased IPSC decay kinetics (Haas and Macdonald, 1999; Bianchi and Macdonald, 2002), and DMV neurons have functional δ-subunit-containing GABA_A_ receptors at GABAergic synapses (Boychuk et al., 2015). Interestingly, not only was the GABA_A_ receptor’s δ-subunit in part required for the augmentation of decay time, but LtAllo also conferred greater functional expression of the δ-subunit. Although qualitative in nature, the expression of the GABA_A_ receptor’s δ-subunit was increased in DMV neurons after LtAllo exposure. Additionally, repeated application of the δ-subunit agonist, THIP, under nACSF conditions resulted in attenuated GABAergic signaling while Allo exposure resulted in a unique potentiation of repeated THIP responses. Therefore, Allo exposure likely recodes inhibitory signaling allowing for otherwise similar inhibitory signaling to encode larger inhibition. Importantly, the lengthening of decay time during LtAllo was not associated with larger phasic GABAergic current amplitude, making it unlikely that a large increase in total GABA_A_ receptor number occurred, but rather receptor subunits are reorganized to increase the expression of the δ-subunit.

The present results confirm that Allo induces augmented inhibition through activation of PKC signaling which requires the δ-subunit. However, the exact relationship between these three cellular components requires significantly more investigations. Since the transmembrane domain of the GABA_A_ receptor contains multiple binding sites for neurosteroids that do not mediate changes in GABA_A_ receptor conductance upon the immediate presence of neurosteroids (Chen et al., 2019), it is possible that the DMV prominently contains GABA_A_ receptor subunit arrangements that confer increased GABAergic currents through less traditional allosteric binding sites. Therefore, Allo could initiate this intracellular signaling cascade by directly binding to the GABA_A_ receptor in the DMV neuron’s plasma membrane. While this binding does not mediate acute increases in receptor pore opening, it could signal changes within the intracellular compartment to activate signal transduction pathways and this dissociation from direct activation of the GABA_A_ receptor was suggested previously (Fancsik et al., 2000). However, more recent evidence identified a neurosteroid signaling pathway through actions on metabotropic progesterone receptors that also facilitate protein kinase-dependent increases in GABAergic neurotransmission (Parakala et al., 2019). Therefore, future investigations into the role of PKC-dependent Allo-induced inhibition of DMV neurons is warranted and should attempt to resolute which receptor(s) are responsible for neurosteroid activation of intracellular PKC activity.

Although subtle changes in GABA_A_ receptors at extrasynaptic locations cannot be completely ruled out, there was no difference in the amplitude of tonic current after Allo exposure under any condition tested. Therefore, Allo-induced modulation of GABA_A_ receptor activity under these conditions is specific to receptors located at the synapse. This spatial specificity of Allo’s actions across neuronal compartments was surprising given the relatively large conductance of tonic currents in DMV neurons (Ferreira et al., 2001; Gao and Smith, 2010a). Additionally, blocking the production of neurosteroids in female mice abolished estrous-cycle dependent changes in GABA_A_ receptor tonic, but not phasic, currents in DMV neurons (Littlejohn et al., 2019). Although sex differences do exist in Allo concentrations in the brainstem (Sze et al., 2018), Allo was specifically chosen here since previous reports demonstrated minimal sex differences compared to other neurosteroids (Wilson and Biscardi, 1997). Therefore, either sex differences do exist in the effect of Allo in the DMV or more prolonged exposure (e.g., hours to days) are need to alter receptor activity at extrasynaptic locations. Additionally, the actions of Allo can vary significantly depending on factors like ambient GABA concentration, and the presence of other GABA receptors/modulators (Khatri et al., 2019). Thus, the DMV might be a unique region of action for neurosteroids where multiple factors, notably timing, are critical for determining neurosteroid-dependent activity on the GABA_A_ receptor.

In addition to Allo effects on GABA_A_ receptors in DMV neurons, our data further implicate LtAllo signaling in the modulation of *presynaptic* inhibitory signaling to the DMV. During LtAllo, there was a significant increase in IPSC frequency which was TTX-insensitive, and likely occurring directly at GABAergic synapses in the DMV. Previous reports suggest that Allo produces a significant reduction in vagal afferent signaling to the NTS (Kim et al., 2018). Reduced vagal afferent activity via deafferentation can influence signal transduction pathways in presynaptic GABAergic terminals resulting in increased state-dependent GABAergic signaling to DMV neurons (Browning et al., 2006; Browning and Travagli, 2009). Indeed, the activation of PKC through PMA recapitulated LtAllo-induced increases in IPSC frequency, suggesting a novel influence of PKC to increase inhibition in vagal circuitry. Additionally, LtAllo failed to influence IPSC frequency in mice lacking the δ-subunit making both PKC and the δ-subunit critical for the influence of LtAllo on presynaptic GABA release. Taken together, these data suggest that PKC activity is likely low at GABAergic presynaptic terminals and Allo decreases vagal afferent terminal activity resulting in the activation of PKC. PKC activation is then sufficient to increase the release of GABA quanta.

Interestingly, both the effects of LtAllo on decay time and frequency correlated with more rostrally located DMV neurons. Topographically, the DMV is traditionally defined by medial-lateral columnar organizations of different vagal branches (Fox and Powley, 1985). Organ projection target does not follow such stringent rules (Berthoud et al., 1991), and while electrophysiological parameters can be unique to projection class, these parameters are overlapping (Browning et al., 1999, 2005). However, rostral to caudal topographical specificity to drug responses have been reported (Rossiter et al., 1990; Krowicki et al., 1997; Takahashi and Owyang, 1997; Zheng et al., 1999). Future studies should investigate the projection class and function of these more rostrally located neurons in relation to neurosteroid activity.

Regardless of its location of action, Allo activity, and neurosteroids in general, likely represent a state modulator in vagal circuits, especially given the timing of changes identified in the present study. Two important physiological states typically associate with large changes in neurosteroid concentration: pregnancy and stress. Both conditions require unique metabolic and cardiovascular responses. Allo has been suggested as a mechanism for baroreflex resetting associated with pregnancy (Heesch, 2011; Kim et al., 2018) and neurosteroid sensitivity is regulated during pregnancy through differential regulation of intracellular kinases activity (Koksma et al., 2003). Stress also increases levels of neurosteroids (including Allo) within the brain (Purdy et al., 1991; Sze et al., 2018), and stress alters brainstem-mediated homeostatic reflexes (Scheuer, 2010). The elevated neurosteroid signaling during these conditions could serve to recode inhibitory signaling to differentially integrate physiologically relevant stimuli. The DMV, then, is uniquely positioned for modulating integrative homeostatic responses to these conditions because of its ability to influence both metabolic and cardiovascular function. However, abnormally elevated neurosteroid activity could also dysregulate homeostatic circuits since for example obesity and polycystic ovary syndrome associate with high neurosteroid signaling (Holmberg et al., 2018). Therefore, the DMV is a unique, yet understudied, brain region of neurosteroid responses that could contribute to modulation of physiological homeostasis.

## Data Availability Statement

The raw data supporting the conclusions of this article will be made available by the authors, without undue reservation.

## Ethics Statement

The animal study was reviewed and approved by the University of Texas Health Science Center at San Antonio Animal Care and Use Committees.

## Author Contributions

ELL and CRB designed, executed, analyzed all the experiments in the present manuscript, prepared, and wrote the present manuscript. Both authors contributed to the article and approved the submitted version.

## Conflict of Interest

The authors declare that the research was conducted in the absence of any commercial or financial relationships that could be construed as a potential conflict of interest.

## Publisher’s Note

All claims expressed in this article are solely those of the authors and do not necessarily represent those of their affiliated organizations, or those of the publisher, the editors and the reviewers. Any product that may be evaluated in this article, or claim that may be made by its manufacturer, is not guaranteed or endorsed by the publisher.

## Supplementary Material

Supplementary Figure 1 Application of vehicle does not alter GABA_A_ receptor IPSCs. **(A)** Representative sIPSC trace from DMV neurons during acute (10 min superfusion in recording chamber) treatment with Veh (0.01% DMSO; Veh; beige trace) and under control conditions without DMSO (aCSF; teal trace). Line and arrow indicate the section expanded below. **(B)** Mean ± SEM of sIPSC decay time during aCSF-treatment (13.5 ± 0.71 ms; *n* = 12 from 8 mice), during Veh-treatment (14.4 ± 0.71 ms; *n* = 14 from 9 mice), and after removal of Veh-treatment (duplicated from Figure 1E) during control conditions. **(C)** Mean ± SEM of sIPSC frequency during aCSF-treatment (2.5 ± 1.0 Hz; *n* = 5 from 4 mice), during Veh-treatment (2.1 ± 0.5 Hz; *n* = 13 from 8 mice), and removal from Veh-treatment (duplicated from Figure 2) during control conditions. Overlaid points denote individual neuronal responses. Significance indicates *p* ≤ 0.05.

Supplementary Figure 2 Allo in the DMV does not modulate tonic GABA_A_ receptor currents. **(A)** Representative sIPSC recording from a DMV neuron during acute (10 min superfusion in recording chamber) treatment with Allo. **(B)** Mean ± SEM of holding currents from DMV neuron during aALLO treatment (56 ± 14 pA during before Veh vs. 46 ± 11 pA; *n* = 7 from 3 mice during aALLO; Paired Student’s *t*-test, *p* = 0.29). **(C)** Representative sIPSC recordings from an individual DMV neuron treated with either LtAllo or Veh. To determine tonic current differences after LtAllo, we examined GABAergic tonic currents by applying BIC (30 μM) and assessing the differences in holding current from pre-BIC to steady-state BIC application as indicated by dashed line. **(D)** Mean ± SEM of BIC-induced tonic current density from LtAllo-treated DMV neurons (1.3 ± 0.3 pA/pF; *n* = 12 from 8 mice) compared to Veh-treated neurons (1.1 ± 0.2 pA/pF; *n* = 10 from 7 mice; unpaired Student’s *t*-test, *p* = 0.52). Tonic currents were also normalized to cell capacitance (tonic current density). There was no statistical difference in cell capacitance between either treatment group (Vehicle 30 ± 3.6 pF vs. Allo 35 ± 3.2 pF; unpaired Student’s *t*-test, *p* = 0.31).
