# Supplementary figures and images for: Protein Kinase C-Dependent Effects of Neurosteroids on Synaptic GABAA Receptor Inhibition Require the δ-Subunit
Source: Front Physiol. 2021 Oct 25;12:742838. doi: 10.3389/fphys.2021.742838 (PMC8573421; doi:10.3389/fphys.2021.742838)

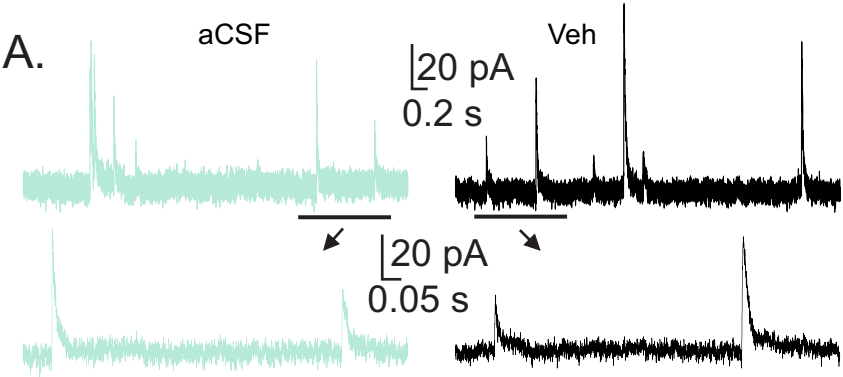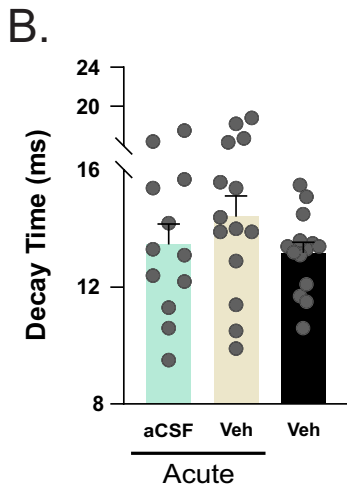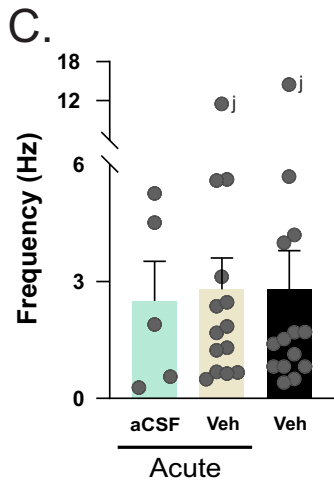

Supplement: Supplementary Figure 1 — Application of vehicle does not alter GABAA receptor IPSCs. (A) Representative sIPSC trace from DMV neurons during acute (10 min superfusion in recording chamber) treatment with Veh (0.01% DMSO; Veh; beige trace) and under control conditions without DMSO (aCSF; teal trace). Line and arrow indicate the section expanded below. (B) Mean ± SEM of sIPSC decay time during aCSF-treatment (13.5 ± 0.71 ms; n = 12 from 8 mice), during Veh-treatment (14.4 ± 0.71 ms; n = 14 from 9 mice), and after removal of Veh-treatment (duplicated from Figure 1E) during control conditions. (C) Mean ± SEM of sIPSC frequency during aCSF-treatment (2.5 ± 1.0 Hz; n = 5 from 4 mice), during Veh-treatment (2.1 ± 0.5 Hz; n = 13 from 8 mice), and removal from Veh-treatment (duplicated from Figure 2) during control conditions. Overlaid points denote individual neuronal responses. Significance indicates p ≤ 0.05. [file Image_1.pdf]

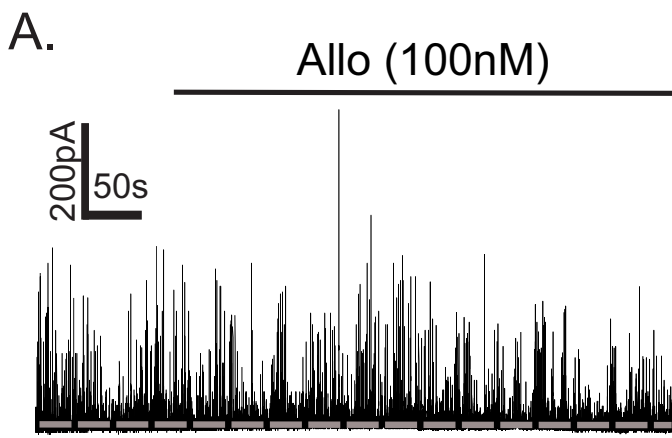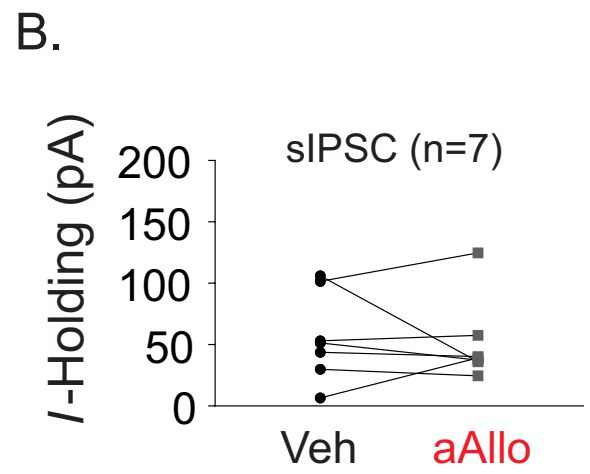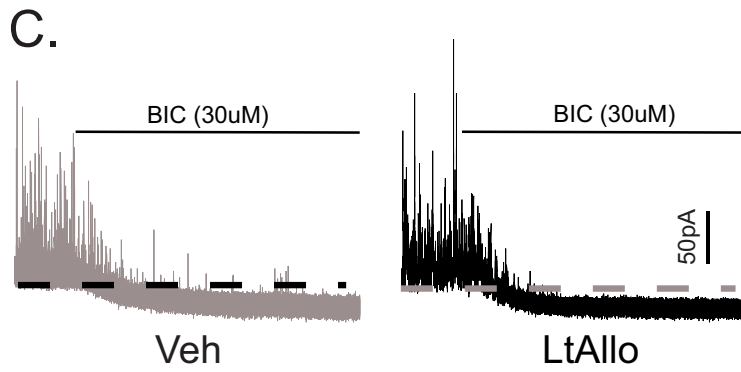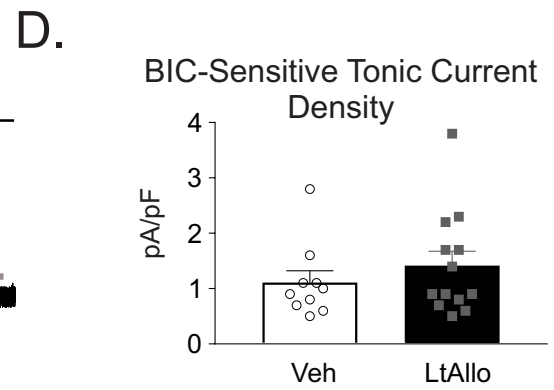

Supplement: Supplementary Figure 2 — Allo in the DMV does not modulate tonic GABAA receptor currents. (A) Representative sIPSC recording from a DMV neuron during acute (10 min superfusion in recording chamber) treatment with Allo. (B) Mean ± SEM of holding currents from DMV neuron during aALLO treatment (56 ± 14 pA during before Veh vs. 46 ± 11 pA; n = 7 from 3 mice during aALLO; Paired Student’s t-test, p = 0.29). (C) Representative sIPSC recordings from an individual DMV neuron treated with either LtAllo or Veh. To determine tonic current differences after LtAllo, we examined GABAergic tonic currents by applying BIC (30 μM) and assessing the differences in holding current from pre-BIC to steady-state BIC application as indicated by dashed line. (D) Mean ± SEM of BIC-induced tonic current density from LtAllo-treated DMV neurons (1.3 ± 0.3 pA/pF; n = 12 from 8 mice) compared to Veh-treated neurons (1.1 ± 0.2 pA/pF; n = 10 from 7 mice; unpaired Student’s t-test, p = 0.52). Tonic currents were also normalized to cell capacitance (tonic current density). There was no statistical difference in cell capacitance between either treatment group (Vehicle 30 ± 3.6 pF vs. Allo 35 ± 3.2 pF; unpaired Student’s t-test, p = 0.31). [file Image_2.pdf]
